# Supplementary material for: Association between thrombocytopenia and 180-day prognosis of COVID-19 patients in intensive care units: A two-center observational study
Source: PLoS One. 2021 Mar 18;16(3):e0248671. doi: 10.1371/journal.pone.0248671 (PMC7972743; doi:10.1371/journal.pone.0248671)
Supplement: S1 Appendix — (PDF) [file pone.0248671.s001.pdf]

电话随访问卷

本问卷旨在调查重症新冠肺炎患者长期预后情况，患者本人或患者家属可能会被询问数个有关于健康状况的问题。所有的信息都可能用于一项探究血小板减少症与重症新冠肺炎患者长期预后及器官功能的回顾性研究。所有私人信息将得到充分的保护，请您如实回答以下问题。

1. 您是否为患者本人？ [单选题] *

○是

○否（转至第 3 题）

2. 您是否愿意参与本次电话问卷调查，并且同意参与一项探究血小板减少症与重症新冠肺炎患者长期预后及器官功能的回顾性研究？ [单选题] *

本研究不会对您的生活和健康产生任何影响，不涉及任何有创操作，仅收集您的医疗信息、实验室检查结果及电话随访信息用于研究。我们承诺您的所有隐私都将得到充分地保护。

○愿意

○不愿意

3. 您是否为患者直系家属，并对患者的病情较为了解？ [单选题] *

○是

○否

4. 您是否愿意参与本次电话问卷调查，并且同意代理患者本人授权，参与一项探究血小板减少症与重症新冠肺炎患者长期预后及器官功能的回顾性研究？ [单选题] *

○是

○否

5. 患者目前是否健在？ [单选题] *

○是

○否（转至第 6 题）

6. 若患者死亡，患者死亡具体时间及死亡原因为？ [单选题] *

○死亡时间 _________________

○死亡原因 _________________

○不详

7. 患者目前处于下列哪一个地点？ [单选题] *

○家里

○医院

○养老院

○其他 _________________

8. 患者目前是否接受额外的氧疗治疗（包括鼻导管吸氧、无创及有创机械通气等）？ [单选题] *

○是

○无

9. 患者是否感觉自己比生病之前更容易乏力？ [单选题] *

○是

○否

10. 患者是否觉得自己比生病之前更容易在运动后感到气喘？ [单选题] *

○是

○否

本次问卷调查收集的信息都将采用匿名化处理，谢谢参与。

祝愿您和您的家人身体健康！

Telephone questionnaire

This questionnaire is designed to investigate the long-term prognosis of critically ill COVID-19 patients. You and (or) your family will be asked few simple questions about your health condition. All the information will be used in a retrospective study on the association of thrombocytopenia and long prognosis and organ function in critically ill COVID-19 patients. All private information will be fully protected. Please answer following questions frankly.

1. Are you the patient himself or herself? *

○Yes

○No （turn to Q3）

2. Are you willing to join this telephone questionnaire, and agree to participate in a retrospective study about the association between thrombocytopenia and long-term prognosis as well as organ function in critically ill COVID-19 patients? *

This study will not have any impact on your life and health. No invasive procedures will be involved. The medical information, laboratory findings and information of this telephone questionnaire will be collected for study purposes. We promise that your privacy will be fully protected.

○Yes

○No

3. Are you the direct family number of the patient, and know his or her condition well? *

○Yes

○No

4. Are you willing to join this telephone questionnaire, and agree to on behalf of the patient to participate in a retrospective study about the association between thrombocytopenia and long-term prognosis as well as organ function in critically ill COVID-19 patients? *

○Yes

○No

5. Is the patient still alive? *

○Yes

○No (turn to Q6)

6. If the patient died, what was the exact time and cause of his or her death? *

○Time _________________

○Cause _________________

○Not clear

7. Where is the patient now? *

○Home

○Hospital

○Nursing home

○Others _________________

8. Dose the patient receive extra oxygen supply treatment (including nasal oxygen inhalation, non-invasive and invasive mechanical ventilation, etc.)? *

○Yes

○No

9. Does the patient feel weaker than before the illness? *

○Yes

○No

10. Dose the patient feel more asthmatic after exercise than before the illness? *

○Yes

○No

All the information will be anonymous processed. Thanks for your participation. Wish you good health!
