# Supplementary figures and images for: Association between thrombocytopenia and 180-day prognosis of COVID-19 patients in intensive care units: A two-center observational study
Source: PLoS One. 2021 Mar 18;16(3):e0248671. doi: 10.1371/journal.pone.0248671 (PMC7972743; doi:10.1371/journal.pone.0248671)

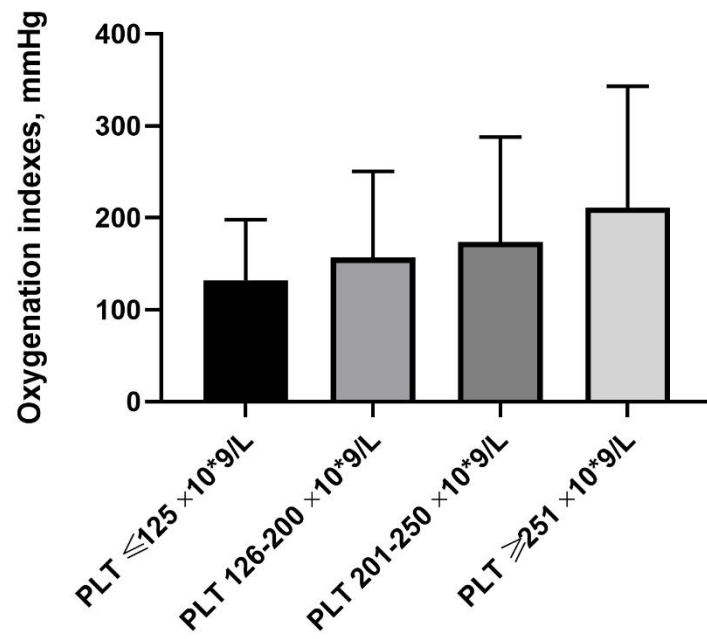

Fig 1. The platelet counts and oxygenation indexes at admission

Supplement: S1 Fig — (PDF) [file pone.0248671.s003.pdf]

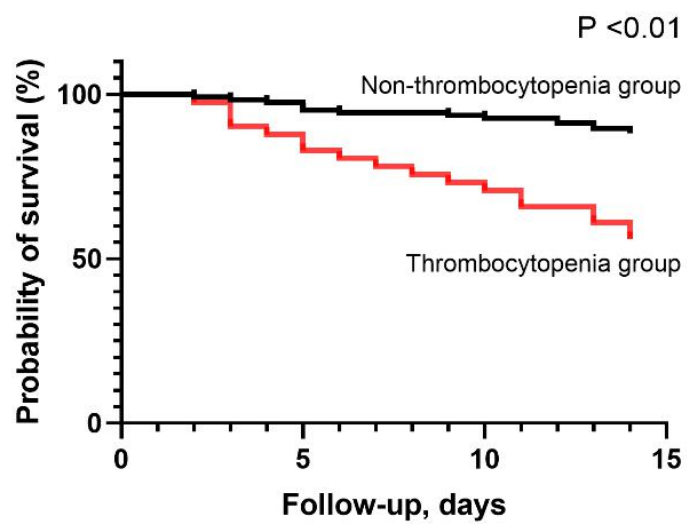

Fig 1. 14-day Survival Curve in Patients with and without Thrombocytopenia.

Supplement: S2 Fig — (PDF) [file pone.0248671.s004.pdf]
