# Supplementary material for: Association between thrombocytopenia and 180-day prognosis of COVID-19 patients in intensive care units: A two-center observational study
Source: PLoS One. 2021 Mar 18;16(3):e0248671. doi: 10.1371/journal.pone.0248671 (PMC7972743; doi:10.1371/journal.pone.0248671)
Supplement: S1 Table — (PDF) [file pone.0248671.s005.pdf]

Table 1. Subsequent changes of organ function.

| Variables | Days | Survival group | Non-survival group | P value |
|---|---|---|---|---|
| Platelet count | 1 | 209.00 [149.75-257.00] | 172.00 [121.00-223.00] | <0.01 |
| | 3 | 186.00 [138.75-261.00] | 166.00 [113.50-245.75] | 0.07 |
| | 7 | 233.000 [160.50-293.50] | 132.00 [95.25-237.50] | <0.01 |
| six-point ordinal scale | 1 | 4.00 [3.00-4.00] | 4.00 [3.00-5.00] | 0.06 |
| | 3 | 4.00 [3.00-4.00] | 4.00 [4.00-5.00] | <0.01 |
| | 7 | 3.50 [3.00-4.00] | 5.00 [4.00-5.00] | <0.01 |
| Oxygenation index | 1 | 135.75 [89.53-238.14] | 113.20 [86.90-202.80] | 0.36 |
| | 3 | 201.30 [122.48-286.33] | 151.70 [91.65-224.38] | 0.03 |
| | 7 | 212.00 [146.82-295.84] | 117.20 [82.80-182.20] | <0.01 |
| SOFA | 1 | 3.50 [2.00-5.00] | 5.00 [3.00-7.00] | <0.01 |
| | 3 | 4.00 [3.00-5.00] | 5.00 [4.00-7.00] | <0.01 |
| | 7 | 3.00 [2.00-5.25] | 5.00 [3.00-8.00] | <0.01 |
| Creatinine, μmol/L | 1 | 64.40 [53.60-89.70] | 75.50 [56.80-134.20] | 0.08 |
| | 3 | 64.30 [49.90-77.00] | 77.50 [52.85-140.45] | 0.07 |
| | 7 | 60.40 [48.70-75.30] | 68.00 [49.70-189.90] | 0.11 |
| blood urea, mmol/L | 1 | 9.80 [6.30—15.10] | 10.10 [6.20-16.44] | 0.89 |
| | 3 | 6.20 [4.35-8.60] | 9.30 []5.79-16.00] | <0.01 |
| | 7 | 6.50 [4.50-8.40] | 11.01 [6.80-20.60] | <0.01 |
| AST, IU/L | 1 | 31.50 [19.80-55.50] | 40.00 [24.00-65.00] | 0.12 |
| | 3 | 29.50 [18.00-54.25] | 36.50 [22.75-58.50] | 0.22 |
| | 7 | 28.00 [19.00-53.25] | 29.00 [20.75-51.50] | 0.67 |
| ALT, IU/L | 1 | 29.00 [19.00-52.00] | 34.00 [20.00-47.00] | 0.74 |
| | 3 | 33.00 [19.00-61.75] | 32.50 [18.00-49.00] | 0.46 |
| | 7 | 36.50 [20.00-75.25] | 25.00 [13.50-43.50] | 0.01 |
| Lymphocyte percentage, % | 1 | 9.90 [5.50-17.30] | 6.70 [3.60-10.40] | <0.01 |
| | 3 | 12.40 [9.70-18.10] | 5.85 [3.35-10.43] | <0.01 |
| | 7 | 15.35 [11.98-21.50] | 5.40 [2.80-6.70] | <0.01 |
| Procalcitonin, mg/mL | 1 | 0.10 [0.06-0.24] | 0.32 [0.12-1.04] | <0.01 |
| | 3 | 0.10 [0.05-0.18] | 0.39 [0.14-2.10] | <0.01 |
| | 7 | 0.09 [0.05-0.27] | 0.46 [0.14-1.52] | <0.01 |
| PT, seconds | 1 | 12.60 [11.63-13.50] | 13.25 [12.18-15.33] | <0.01 |
| | 3 | 12.7 [11.65-13.38] | 14.20 [12.85-15.85 | <0.01 |
| | 7 | 12.25 [11.50-13.20] | 13.60 [12.53-15.30] | <0.01 |
| APTT, seconds | 1 | 31.60 [27.80-34.70] | 32.00 [27.95-41.50] | 0.11 |
| | 3 | 30.35 [26.55-35.18] | 34.40 [28.35-41.55] | 0.02 |
| | 7 | 30.00 [26.55-36.03] | 35.65 [30.35-42.98] | <0.01 |
| WBC, ×109/L | 1 | 7.32 [5.17-10.08] | 9.59 [7.02-13.40] | <0.01 |
| | 3 | 7.03 [5.01-9.46] | 9.09 [7.27-12.11] | <0.01 |
| | 7 | 6.86 [5.60-8.87] | 11.26 [7.52-13.79] | <0.01 |

| | | | | |
|---|---|---|---|---|
| hs-CTn I, ng/mL | 1 | 0.01 [0.01-0.03] | 0.03 [0.02-0.09] | <0.01 |
| | 3 | 0.02 [001-0.05] | 0.06 [0.03-0.21] | <0.01 |
| | 7 | 0.03 [0.01-0.09] | 0.07 [0.03-0.23] | 0.01 |
