# Supplementary material for: Association between thrombocytopenia and 180-day prognosis of COVID-19 patients in intensive care units: A two-center observational study
Source: PLoS One. 2021 Mar 18;16(3):e0248671. doi: 10.1371/journal.pone.0248671 (PMC7972743; doi:10.1371/journal.pone.0248671)
Supplement: Attachment — Submitted filename: Manuscript Review.odt [file pone.0248671.s007.odt]

Dear Editor,
Thanks again for giving me the opportunity to review the manuscript entitled ‘’ Association between thrombocytopenia and 180-day prognosis of COVID-19 patients in intensive care units: a two-center observational study’’.
The authors did substantial changes in the manuscript. I would advise for accepting the revised version.
Regards.
Anis Chaari (MD)
